# The anti-inflammatory activity of resveratrol in acute kidney injury: a systematic review and meta‐analysis of animal studies

**DOI:** 10.1080/13880209.2022.2132264

**Published:** 2022-10-21

**Authors:** Shangmei Cao, Xiuhong Fu, Shaozhe Yang, Shuifu Tang

**Affiliations:** a Department of Science and Technology Innovation Center, Luohe Central Hospital, The First Affiliated Hospital of Luohe Medical College, Henan Key Laboratory of Fertility Protection and Aristogenesis, Luohe, China; b Department of Obstetrics and Gynaecology, Luohe Central Hospital, The First Affiliated Hospital of Luohe Medical College, Henan Key Laboratory of Fertility Protection and Aristogenesis, Luohe, China; c Department of Reproductive and Genetic Center, Luohe Central Hospital, The First Affiliated Hospital of Luohe Medical College, Henan Key Laboratory of Fertility Protection and Aristogenesis, Luohe, China; d Division of Nephrology, First Affiliated Hospital of Guangzhou University of Chinese Medicine, Guangzhou, China

**Keywords:** Plant-derived agents, anti-inflammatory

## Abstract

**Context:**

Accumulated experimental evidence suggests that resveratrol (RSV) may have an effect on acute kidney injury (AKI) by inhibiting inflammation. However, the credibility of the evidence for this practice is unclear.

**Objective:**

This study investigated the effect of RSV on AKI and the underlying mechanism.

**Methods:**

We searched PubMed, EMBASE, and Web of Science from 2005 to April 2022 for controlled animal trials assessing the effect of conventional resveratrol versus placebo on renal function outcome after AKI. This study was registered within the International Prospective Register of Systematic Reviews (PROSPERO) as number CRD42022329596.

**Results:**

We retrieved 455 studies, 25 studies comprising data of 436 animals that met the inclusion criteria. Our meta-analysis suggested that RSV treatment was significantly associated with lower levels of serum creatinine (Scr) and blood urea nitrogen (BUN). The greatest effects were recorded in low-dose (<20 mg/kg/day) groups rather than in high-dose (> 20 mg/kg/day) groups. For time-response effects, subgroup analysis indicated that intervention duration of RSV can influence the treatment effect, and more beneficial effects were observed when studies had a drug administration time of <2 weeks.

**Discussion and conclusions:**

This systematic review of animal AKI studies showed a consistently favourable effect of RSV as compared to placebo on renal function outcomes that increased with lower TNF-α, IL-6, and IL-1β. RSV has a more beneficial effect on SA-AKI animal models than the others. When the RSV intervention dose was low (< 20 mg/kg/day) and the intervention time was <2 weeks, more benefits could be observed.

## Introduction

Acute kidney injury (AKI) is a worldwide public health issue, estimated to result in millions of deaths annually (Kellum et al. 2021; Zankar et al. 2021). The sudden increase of serum creatinine level in a short time (generally within 7 days) (a marker of renal excretion function) and the decrease of urine volume (a quantitative marker of urine production) are the markers of acute renal injury, indicating the loss of renal function. If the acute renal injury continues unabated (the duration is usually 3 months), it will develop into chronic renal injury (Kang et al. 2021; Feng et al. 2022; Levey 2022). AKI describes a sudden loss of kidney function that is determined on the basis of increased serum creatinine levels (a marker of kidney excretory function) and reduced urinary output (oliguria) (a quantitative marker of urine production) and is limited to a duration of 7 days. The pathophysiology and diagnosis of AKI are applied to a variety of settings, such as infections, sepsis, surgery, trauma, nephrotoxic medications, and heart disease, including its long-term consequences (Farrar 2018).

Resveratrol (RSV), the main effective component of traditional Chinese medicine *Reynoutria japonica* Houtt. (Polygonaceae), is a natural activator of SIRT1, and has antioxidation and anti-aging effects. Resveratrol can alleviate a variety of renal injuries including renal injury caused by ischemia-reperfusion (IRI) (Buys-Gonçalves et al. 2020; Wang et al. 2020; Hemsinli et al. 2021), drug-induced kidney injury (DKI) (Xiao et al. 2021) and sepsis-associated acute kidney injury (SA-AKI) (Wang et al. 2021). However, one study also indicated that RSV does not protect from ischemia-induced acute kidney injury in rat model (Bienholz et al. 2017).

Therefore, in view of whether RSV can significantly affect AKI renal function, it is necessary to conduct a systematic evaluation to understand the exact therapeutic effect of RSV on AKI caused by what causes, as well as the best intervention dose and time. In this study, we systematically evaluate the therapeutic value of RSV on AKI caused by different factors, so as to understand the therapeutic effect of RSV on AKI and its anti-inflammatory mechanism.

## Methods

This review used the Preferred Reporting Items for Systematic Review and Meta-Analyses statement. The methodological quality of animal studies was assessed based on the Systematic Review Centre for Laboratory animal Experimentation Risk of Bias (SYRCLE’s RoB) tool. The meta-analysis was performed based on the Cochrane Handbook for Systematic Reviews of Interventions by using Review Manager 5.3 (the Cochrane Collaboration). The International Prospective Register of Systematic Reviews registration code was CRD42022329596.

### Article selection

We searched PubMed, EMBASE, and Web of Science scholar electronic databases. We included only English-language articles published from 2005 to April 2022. The databases were searched using the following search terms in titles and abstracts (also in combination with MESH terms): (mice OR rat) AND (Acute kidney injury OR AKI) AND (resveratrol OR trans-resveratrol OR cis resveratrol). The electronic search was complemented by manual searching of the references of the included publications. The studies were included if they met specific inclusion and exclusion criteria (Table 1). No restriction in terms of year of publication was applied.

**Table 1.** Inclusion and exclusion criteria.

Inclusion criteria	Exclusion criteria
1. All animal models with AKI	1. Animals with co-morbidity, clinical trials, and in vitro models
2. RSV with all dosage and duration	2. RSV without batch number
3. Same solvent (e.g., water and saline), no intervention, etc.	3. Other preparation of RSV
4. Serum creatinine (Scr), and blood urea nitrogen (BUN) were the primary outcomes, IL-1β, IL-6, TNF-α were the secondary outcomes	4. Case studies, cross over studies, and studies without a separate Control group
5. Controlled studies with a separate control group	5. Not an original full research paper
6. Language: English	6. Duplicate publication
	7.Studies without full text

### Study selection and data extraction

From all eligible publications, the following data, including the first author, year of publication, sample size, weight, species, animal models of AKI in the experimental group, source of controls, intervention duration and dose, and outcome measures (Scr, BUN, TNF-α, IL-6, IL-1β), were carefully extracted by two authors (SY and SC) independently. Inconsistencies were resolved after discussion, and a consensus was reached for all extracted data. Any disagreements between reviewers over the data extraction were resolved through discussion with a third reviewer.

### Quality assessment

The risk of bias was assessed with the risk of bias tool of the Systematic Review Centre for Laboratory animal Experimentation (SYRCLE) for randomised trials. The SYRCLE’s tool for animal experiments is based on types of bias as follows: (1) Baseline characteristics (selection bias); (2) Random sequence generation (selection bias); (3) Random housing (performance bias); (4) Allocation concealment (selection bias); (5) Random outcome assessment (detection bias); (6) Incomplete outcome data (attrition bias); (7) Random outcome assessment; (8) Blinding (detection bias); (9) Selective outcome reporting (reporting bias); (10) Other sources of bias (other). The results of the assessment are ‘yes’, ‘no’, and ‘unclear’, representing ‘low risk of bias’, ‘high risk of bias’, and ‘insufficient details have been reported to assess the risk of bias properly’. Two reviewers performed quality the assessment independently, and discrepancies were discussed with a third reviewer.

### Statistical analysis

All the outcome measures were continuous data (e.g., BUN and Scr), so we performed random-effects meta-analyses of continuous data with standardised mean differences (SMD) and their 95% confidence intervals (95% CI), a *p*-value <0.05 was considered to be statistically significant. A random-effect model was implemented to calculate the pooled results. Heterogeneity was assessed using a Q statistic (considered significant heterogeneity among the studies if *p* value < 0.10) and an *I-*squared (*I*^2^) value (Higgins and Green 2011). When heterogeneity of studies was significant, the DerSimonian and Laird random-effects model (DerSimonian and Laird 1986) was performed to calculate the pooled ORs. Otherwise, the Mantel–Haenszel fixed-effects model was used (Mantel and Haenszel 1959). We performed the sensitivity analysis to explore heterogeneity when significant heterogeneity was detected. Subgroup analysis was performed to investigate the possible sources of heterogeneity based on the following variables if there were adequate studies: dosage (low < 20 mg/kg/day; high ≥ 20 mg/kg/day), intervention duration (<2 weeks; ≥ 2 weeks), AKI models (ischemia AKI; SA-AKI; DKI), and species (rats; mice). Sensitivity analysis was conducted to evaluate whether a single study affects the overall effect sizes by removing one study at each stage. Moreover, publication bias was evaluated quantitatively using Begg’s (Begg and Mazumdar 1994) and Egger’s tests (Egger et al. 1997). Significant publication bias was indicated if the *p*-value < 0.05. Review Manager 5.3 (the Cochrane Collaboration) and STATA 16.0 (Stata Corporation) were used for the analysis.

## Results

### Study inclusion

**Figure 1.**
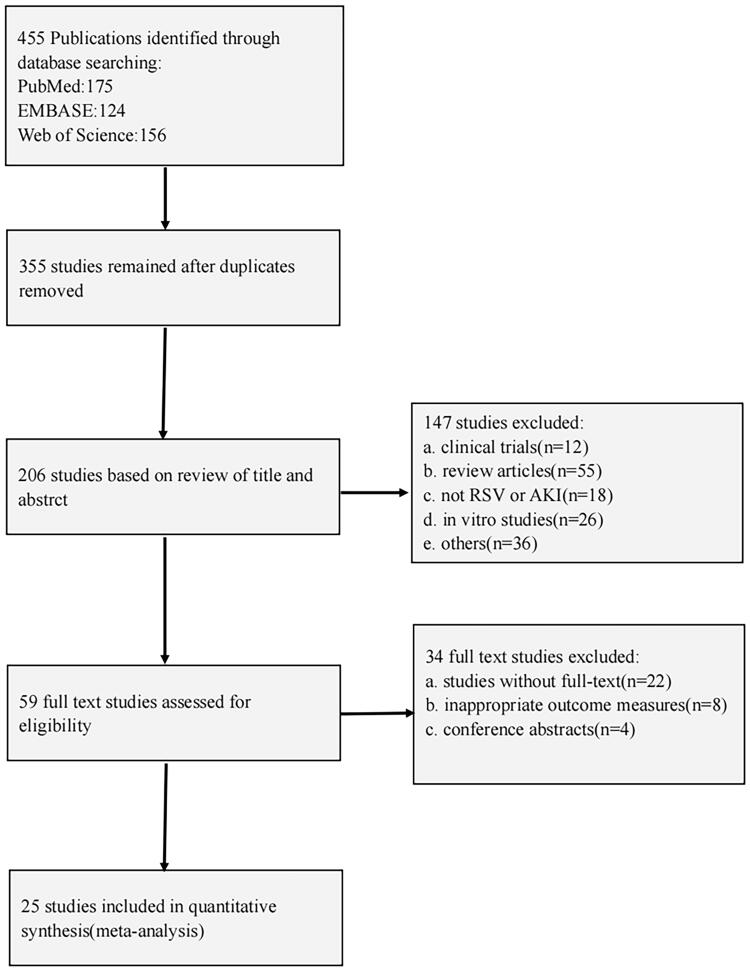
Flow diagram of the study selection process for this review.

The process of study selection is shown in Figure 1. The electronic search and other sources identified 455 animal studies, after removing duplicates, 355 publications remained, of which 206 were excluded after the title and abstract reading. Thus, a total of 25 full-text articles were assessed for eligibility.

### Study characteristics

The 25 selected studies on animals were published between 2005 and 2021. The number of all animals in the experimental group was 218 and that in the control group was 218. The animal species included mice and rats, 3 studies (12%) used mice, and 22 studies (88%) used rats. The weight of rats ranged from 150 to 250 g in most of these studies, only one study reported the rat weight 40–60 g was used (Wang et al. 2018), the weeks old of rats ranged from 6 to 9 weeks. The weight of mice ranged from 150 to 200 g, which weeks old ranged from 7 to 40 weeks. There were three animal models in these studies, IRI (40%), DKI (32%), and SA-AKI (28%).

Two levels (low and high) of RSV doses were implemented in these studies and the dose ranged from 3 to 100 mg/kg/day. Control groups mainly used the same solvent saline. The intervention duration consisted of <2 weeks and ≥2 weeks, and the duration ranged from 3 h to 12 weeks. The intervention duration was ≥2 weeks in 9 studies (36%) and <2 weeks in 16 studies (64%). The main characteristics and main results of the included studies are summarised in Table 2.

**Table 2.** Characteristics of the included studies.

Study year	*N*	Species	Weight (g/weeks)	Types of AKI	RSV dose (mg/kg/day)	Duration	Control	Outcome index
Vikas et al. (2005)	7	Male SD rat	200–250 g	IRI	5	24h	Saline	1. BUN 2. Scr
Vikas C et al. (2006)	7	Male Wistar rat	150–200 g	IRI	5	24h	Saline	1. BUN 2. Scr
Vikas et al. (2006)	7	Male SD rat	150–200 g	DKI (glycerol)	10	24h	Saline	1. BUN 2. Scr
Cátia et al. (2008)	6	Male Wistar rat	180–220 g	DKI (cisplatin)	25	2days	Saline	1. BUN 2. Scr
Hideyuki et al. (2014)	8	Male SD rat	6 weeks	IRI	5	24h	Saline	1. BUN 2. Scr
Hussain et al. (2016)	4	Male Wistar rat	190–200 g	DKI(aluminum chloride)	20	40days	Saline	1. BUN 2. Scr
QIUFA et al. (2016)	7	Male Wistar rat	180–200g	DKI (cisplatin)	10	2days	Saline	1. BUN 2. Scr
Y Gan et al. (2016)	10	Male SD rat	150–200 g	SA-AKI	10	72h	Saline	1. BUN 2. Scr 3. TNF-α 4. IL-1β
Honga et al. (2017)	6	Male C57BL/6J mice	7–10 weeks	DKI (contrast media)	30	24h	Saline	1. BUN 2. Scr
Nian et al. (2017)	5	Male SD rat	180–200g	SA-AKI	20	12h	Saline	1. BUN 2. Scr 3.IL-6 4. TNF-α 5. IL-1β
Mostafa et al. (2018)	6	Male Wistar rat	200–250 g	DKI (cisplatin)	30	14days	Saline	1. BUN 2. Scr
Seldag et al. (2019)	9	Male Wistar rat	220–250 g	DKI (cyclosporine)	10	14days	Saline	1. BUN 2. Scr
Gabriela et al. (2020)	10	Male Wistar rat	9 weeks	IRI	30	28days	Saline	1. BUN 2. Scr
Hemsinli et al. (2020)	8	Male SD rat	3–4 months	IRI	10	3h	Saline	1. BUN 2. Scr
Li et al. (2020)	5	Male SD rat	220–250 g	IRI	30	7days	Saline	1. BUN 2. Scr
Gabriela et al. (2015)	10	Male Wistar rat	9 weeks	IRI	30	28days	Saline	1. BUN 2. Scr
Sun et al. (2016)	10	Male SD rat	150–200 g	SA-AKI	20	28days	Saline	1. BUN 2. Scr
Qin et al. (2017)	6	Fisher and Lewis rats	150–220 g	IRI	30	12weeks	Saline	1. BUN 2. Scr
Joseph et al. (2017)	9	Male C57/BL6 mice	39–40 weeks	SA-AKI	10	48h	Saline	1. BUN 2. Scr
Liang et al. (2020)	8	Female C57BL/6 mice	150–200 g	SA-AKI	100	20h	Saline	1. BUN 2. Scr 3.IL-6 4. TNF-α 5. IL-1β
YAN et al. (2020)	6	Male SD rat	40–60 g	SA-AKI	30	30h	Saline	1. BUN 2. Scr 3. TNF-α 4. IL-1β
Yi-Hsin et al. (2015)	8	Male SD rat	150–200 g	DKI (contrast media)	30	3days	Saline	1. BUN 2. Scr
Anand et al. (2016)	8	Male SD rat	180–230 g	DKI(Nicotine)	8	28days	Saline	1. BUN 2. Scr
Benxi et al. (2017)	8	Male SD rat	250–300 g	DKI(uric acid)	100	21 days	Saline	1. BUN 2. Scr 3.IL-6 4. TNF-α
Biao et al. (2017)	40	Male SD rat	150–200 g	SA-AKI	3	12h	Saline	1. BUN 2. Scr 3.IL-6 4. TNF-α 5. IL-1β

Abbreviations: BUN: blood urea nitrogen; DKI: drug-induced kidney injury; IL- 1β: interleukin-1β; IL-6: interleukin-6; IRI: ischemia-reperfusion injury; SA-AKI: sepsis-associated acute kidney injury; Scr: serum creatinine; SOD: superoxide dismutase; TNF-α: tumour necrosis factor-α.

### Study quality

There were 13 studies (52%) that reported the random allocation to experimental and control groups. The distribution of relevant baseline levels and random outcome assessment was not mentioned in all studies. All these studies had complete outcome data and reported expected outcomes. As for other sources of bias, 17 studies (68%) stated that there was no conflict of interest among the authors, and the remaining 8 studies (32%) did not mention it. The risk of bias in individual studies is summarised in Table 3.

**Table 3.** Risk of bias of included studies.

Study year	(1)	(2)	(3)	(4)	(5)	(6)	(7)	(8)	(9)	(10)
Vikas et al. (2005)	Y	U	U	U	U	U	Y	Y	Y	U
Vikas C et al. (2006)	Y	U	U	U	U	U	Y	Y	Y	U
Vikas et al. (2006)	Y	U	Y	U	U	U	Y	Y	Y	U
Cátia et al. (2008)	Y	U	U	U	U	U	Y	Y	Y	U
Hideyuki et al. (2014)	Y	U	U	U	U	U	Y	Y	Y	U
Hussain et al. (2016)	Y	U	Y	U	U	U	Y	Y	Y	U
QIUFA et al. (2016)	Y	U	Y	U	U	U	Y	Y	Y	U
Y Gan et al. (2016)	Y	U	Y	U	U	U	Y	Y	Y	Y
Honga et al. (2017)	Y	U	Y	U	U	U	Y	Y	Y	Y
Nian et al. (2017)	Y	U	U	U	U	U	Y	Y	Y	Y
Mostafa et al. (2018)	Y	U	U	U	U	U	Y	Y	Y	Y
Seldag et al. (2019)	Y	U	Y	U	U	U	Y	Y	Y	Y
Gabriela et al. (2020)	Y	U	Y	U	U	U	Y	Y	Y	Y
Hemsinli et al. (2020)	Y	U	Y	U	U	U	Y	Y	Y	Y
Li et al. (2020)	Y	U	Y	U	U	U	Y	Y	Y	Y
Gabriela et al. (2015)	Y	U	U	U	U	U	Y	Y	Y	Y
Sun et al. (2016)	Y	U	Y	U	U	U	Y	Y	Y	Y
Qin et al. (2017)	Y	U	U	U	U	U	Y	Y	Y	Y
Joseph et al. (2017)	Y	U	U	U	U	U	Y	Y	Y	Y
Liang et al. (2020)	Y	U	U	U	U	U	Y	Y	Y	Y
YAN et al. (2020)	Y	U	U	U	U	U	Y	Y	Y	Y
Yi-Hsin et al. (2015)	Y	U	Y	U	U	U	Y	Y	Y	Y
Anand et al. (2016)	Y	U	Y	U	U	U	Y	Y	Y	Y
Benxi et al. (2017)	Y	U	Y	U	U	U	Y	Y	Y	U
Biao et al. (2017)	Y	U	U	U	U	U	Y	Y	Y	Y

SYRCLE RoB: Systematic Review Centre for Laboratory Animal Experimentation Risk of Bias; (1) random sequence generation (2) baseline characteristics (3) allocation concealment (4) random housing (5) blinding (performance bias) (6) random outcome assessment (7) blinding (detection bias) (8) incomplete outcome data (9) selective outcome reporting (10) other sources of bias. N: no (high risk of bias); U: unclear (unclear risk of bias); Y: yes (low risk of bias).

### Effect of resveratrol on blood urea nitrogen

**Figure 2.**
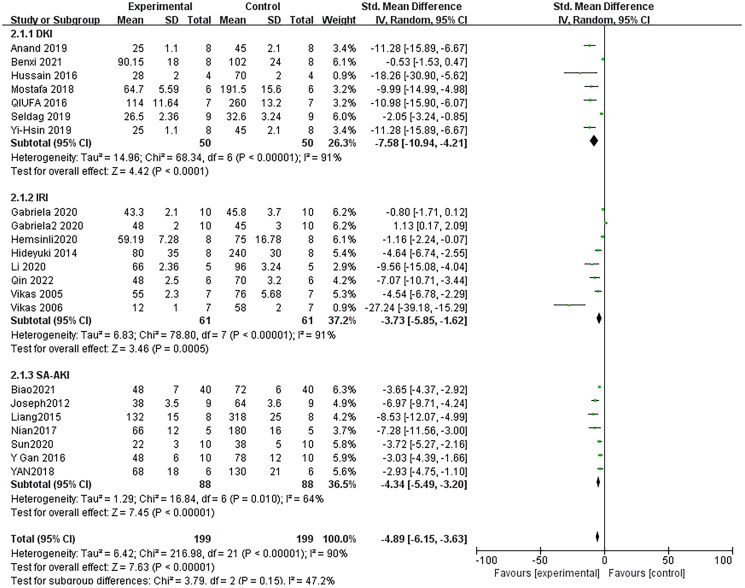
Pooled estimate of BUN with RSV. *Note*: DKI: drug-induced kidney injury; IRI: ischemia-reperfusion injury; SA-AKI: sepsis-associated acute kidney injury.

**Figure 3.**
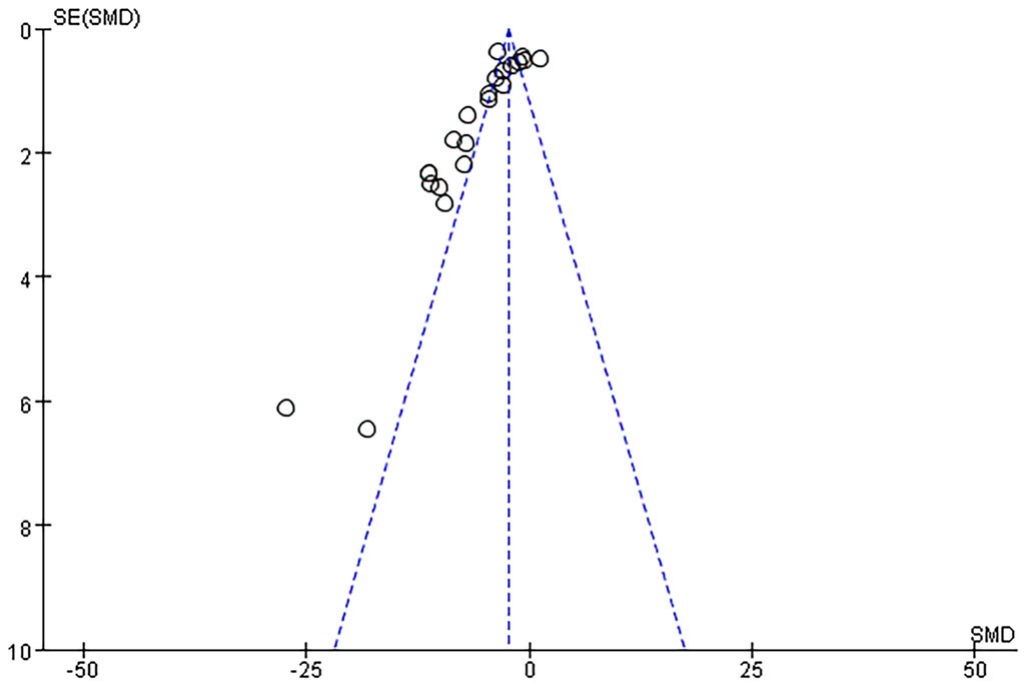
Funnel plot for the effects of RSV on BUN.

Seventeen pair-wise comparisons reported the influence of RSV on BUN. The pooled results suggested that RSV could significantly decrease BUN level compared with the control group [*n* = 398, SMD = −4.89, 95% CI (−6.15, −3.63), *p* < 0.00001; Heterogeneity: *X*^2^ = 216.98, *p* < 0.00001; *I*^2^ = 90%, Figure 2]. The included studies were stratified according to variables including AKI models, better therapeutic effects were observed when studies used SA-AKI models [*n* = 176, SMD = −4.34, 95% CI (−5.49, −3.20), *p*< =0.01; Heterogeneity: *X*^2^ = 16.84, *p* < 0.00001; *I*^2^ = 64%, Figure 2]. Test for subgroup differences, *P > 0.05*, There was no significant difference between the groups, and the comparison between the groups was statistically significant. Funnel plots showed asymmetry for the effects of RSV on BUN (Figure 3), while the result of Egger’s test was statistically significant [intercept: −5.53, 95% CI (−6.30, −2.84); *p* = 0.000].

### Effect of resveratrol on serum creatinine

**Figure 4.**
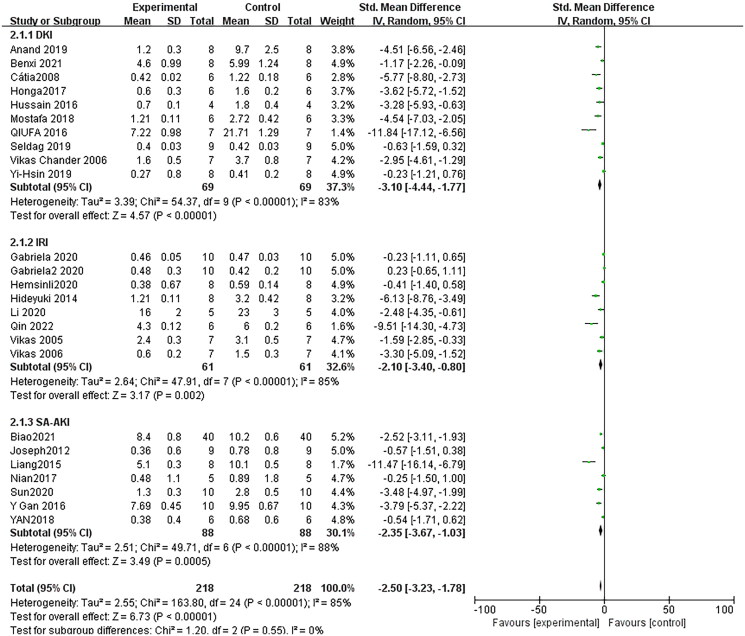
Pooled estimate of Scr with RSV.

**Figure 5.**
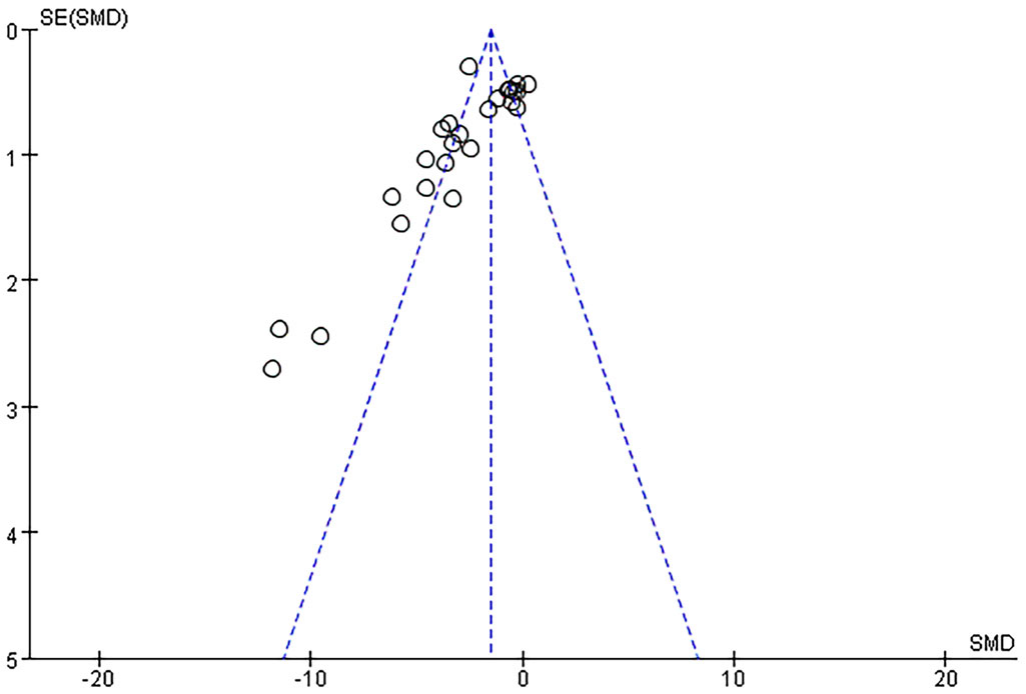
Funnel plot for the effects of RSV on Scr.

Combining effect sizes from 27 pair-wise comparisons, a significant reduction in Scr level was observed after RSV administration, compared to that in the control group [*n* = 436, SMD = −2.35, 95% CI (−3.23, −1.78), *p* < 0.00001; Heterogeneity: *X*^2^ = 163.80, *p* < 0.00001; *I*^2^ = 85%, Figure 4]. Subgroup analysis was performed according to AKI models, more beneficial effects were observed when studies applied DKI models [*n* = 138, SMD = −3.10, 95% CI (−4.44, −1.77), *p* < 0.00001; Heterogeneity: *X*^2^ = 54.37, *p* < 0.00001; *I*^2^ = 83%, Figure 4]. Test for subgroup differences, *p > 0.05*, There was no significant difference between the groups, and the comparison between the groups was statistically significant. Furthermore, visual inspection of funnel plots showed asymmetry for the effects of RSV on Scr (Figure 5), and the result of Egger’s test was statistically significant [intercept: −4.82, 95% CI (−6.17, −2.46); *p* = 0.000].

### Effect of resveratrol on TNF-α

**Figure 6.**
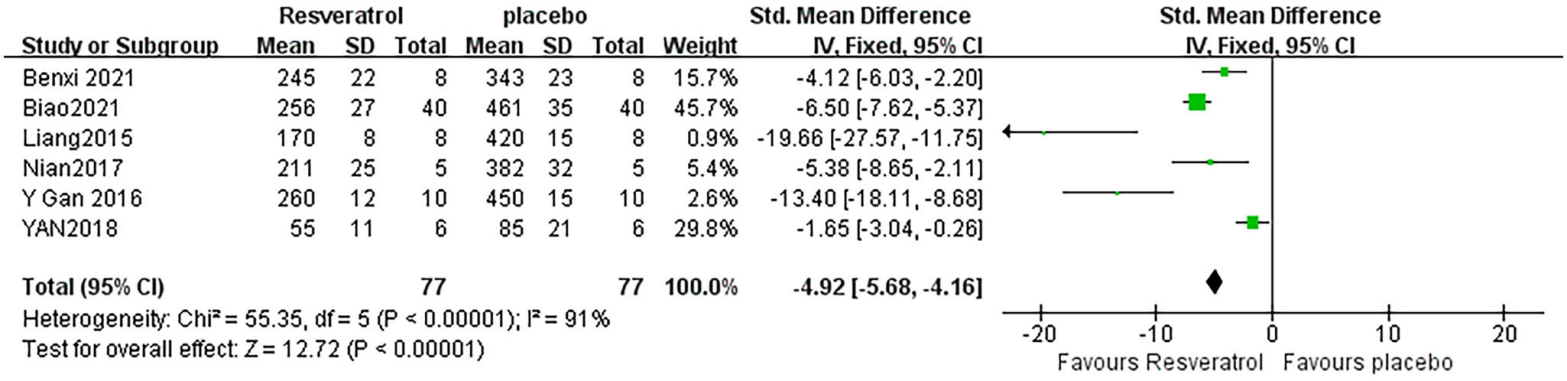
Pooled estimate of TNF-α with RSV.

Combining effect sizes from six pair-wise comparisons, there was a significant decrease in the level of TNF-α was observed after RSV administration, compared to that in the control group [*n* = 154, SMD = −4.92, 95% CI (−5.68, 4.16), *p* < 0.00001; Heterogeneity: *X*^2^ = 55.35, *p* < 0.00001; *I*^2^ = 91%, Figure 6]. Subgroup analysis was not conducted according to species because all included studies applied rat models of SA-AKI. In addition, publication bias was not conducted on TNF- α as less than 10 studies were included.

### Effect of resveratrol on IL-6

**Figure 7.**
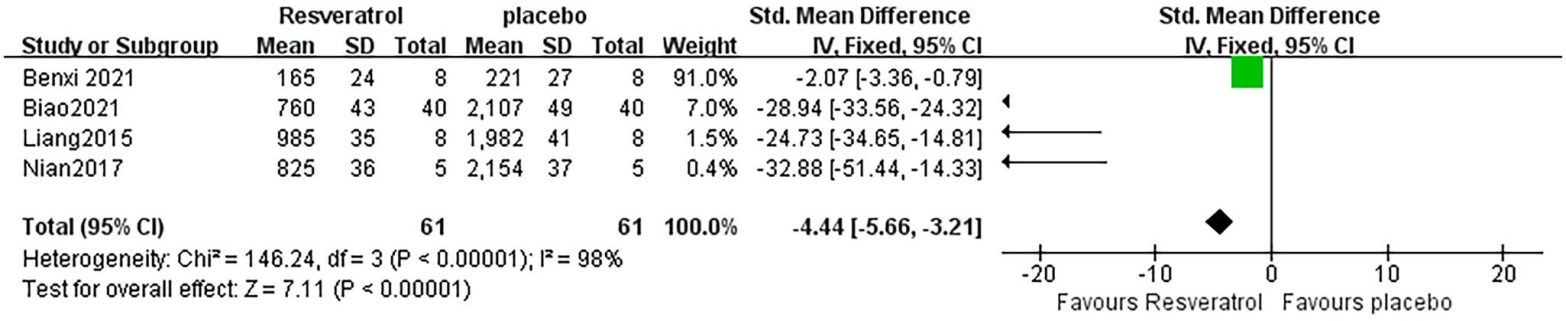
Pooled estimate of IL-6 with RSV.

As for the effect on IL-6, 4 pair-wise comparisons mentioned the influence of RSV on this outcome. The pooled effect sizes showed that RSV did significantly decrease IL-6 level compared with the control group [*n* = 122, SMD = −4.44, 95% CI (−5.66, 3.21), *p* < 0.00001; Heterogeneity: *X*^2^ =146.24, *p* < 0.00001; *I*^2^ = 98%, Figure 7]. Furthermore, publication bias was not conducted on IL-6 as less than 10 studies were included.

### Effect of resveratrol on IL-1β

**Figure 8.**
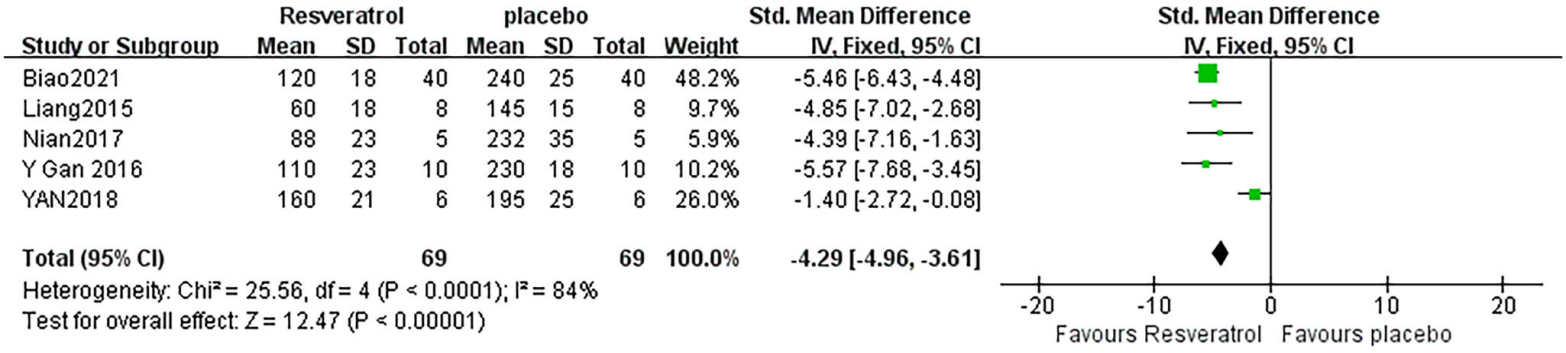
Pooled estimate of IL-1β with RSV.

Effect sizes for IL-1β were pooled from a total of 5 pair-wise comparisons. There was a significant association of RSV with IL-1β level [*n* = 138, SMD = −4.29, 95% CI (−4.96, −3.61), *p* < 0.00001; Heterogeneity: *X*^2^ = 25.56, *p* < 0.00001; *I*^2^ = 84%, Figure 8]. Subgroup analysis was not conducted according to species because all included studies applied rat models of SA-AKI. Publication bias was not performed on IL-6 as less than 10 studies were included.

### Effect of resveratrol on Scr in different dose and duration

**Figure 9.**
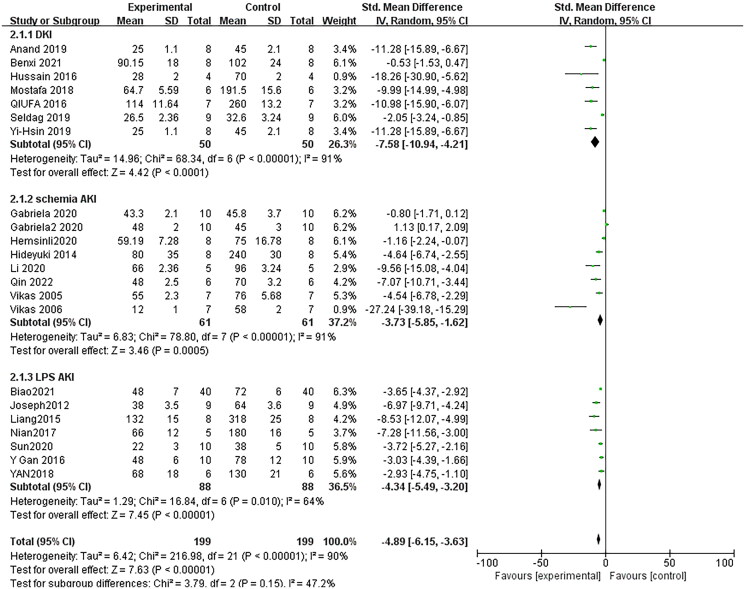
Funnel plot for the effects of RSV on Scr in different dose.

Subgroup analysis was conducted according in different dose and duration showed that effect of RSV in low-dose (<20 mg/kg/day) groups [*n* = 240, SMD = −2.67, 95% CI (−3.23, −1.78), *p* < 0.00001; Heterogeneity: *X*^2^ = 66.60, *p* < 0.00001; *I*^2^ = 85%, Figure 8], in high-dose (≥20 mg/kg/day) groups [*n* = 196, SMD = −2.39, 95% CI (−3.44, −1.34), *p* < 0.00001; Heterogeneity: *X*^2^ = 85.35, *p* < 0.00001; *I*^2^ = 85%, Figure 9].

**Figure 10.**
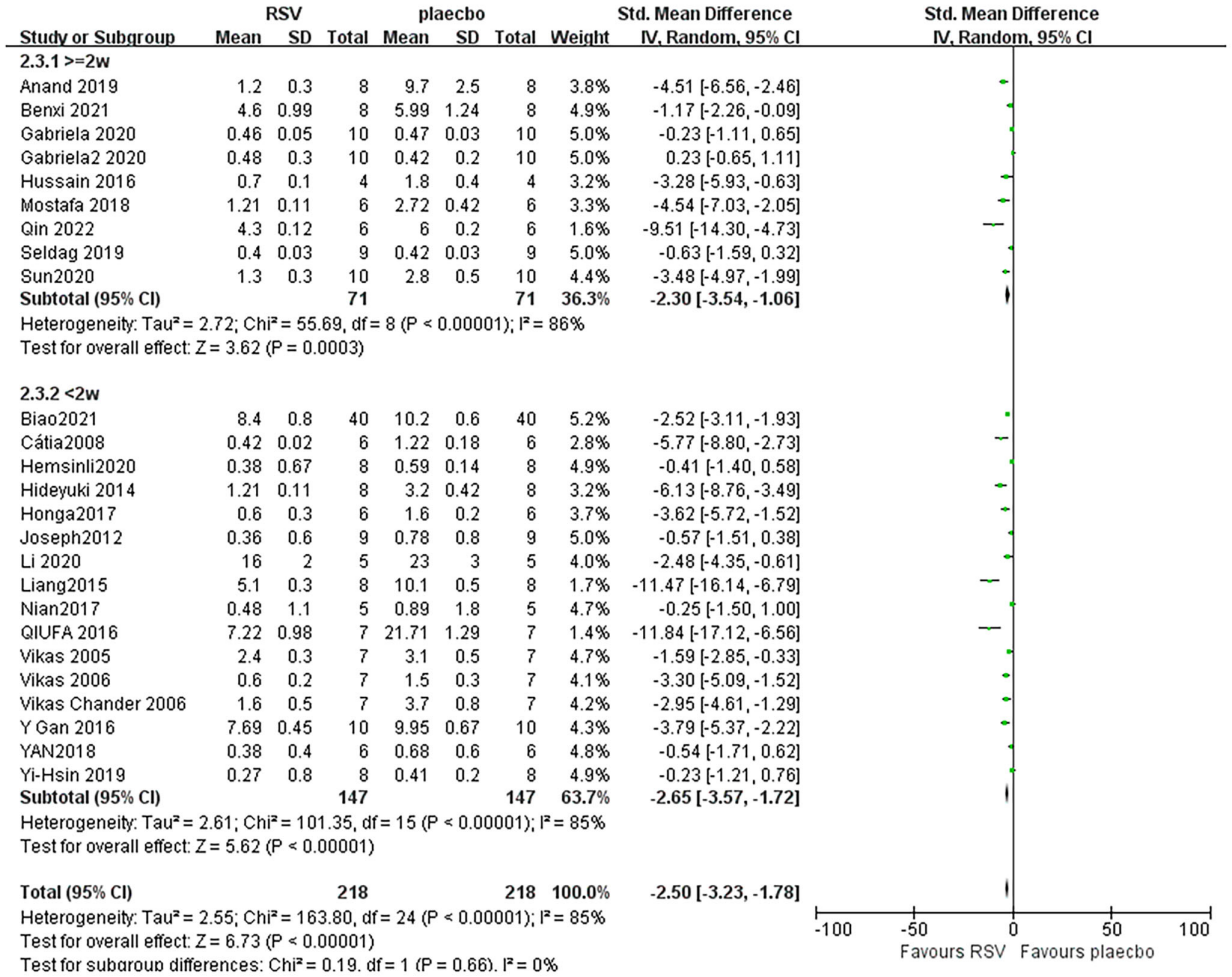
Funnel plot for the effects of RSV on Scr in different duration.

Effect of RSV in administration time of <2 weeks [*n* = 294, SMD = −2.65, 95% CI (−3.57, −1.72), *p* < 0.00001; Heterogeneity: *X*^2^ = 101.35, *p* < 0.00001; *I*^2^ = 85%, Figure 8], administration time of ≥2 weeks [*n* = 142, SMD = −2.30, 95% CI (−3.54, −1.06), *p* < 0.00001; Heterogeneity: *X*^2^ = 55.69, *p* < 0.00001; *I*^2^ = 86%, Figure 10].

### Sensitivity analysis

For Scr, BUN the sensitivity analysis was conducted by removing one study at each stage, and the results indicated that no individual study significantly affected the pooled effect sizes.

Nevertheless, TNF-α, IL-6 and IL-1β were influenced in one study (Wang et al. 2018), and there was a significant association of RSV With TNF-α, IL-6 and IL-1β levels after removing this study [Before sensitivity analysis: TNF-α: SMD = −4.92, 95% CI (−5.68, 4.16), *p* < 0.00001; IL-6: SMD = −4.44, 95% CI (−5.66, 3.21), *p* < 0.00001; IL-1β: SMD = −4.29, 95% CI (−4.96, −3.61), *p* < 0.00001. After sensitivity analysis: TNF-α: SMD = −6.30, 95% CI (−7.21, −5.40), *p* < 0.00001; IL-6: SMD = −28.42, 95% CI (−32.50, −24.33), *p* < 0.00001; IL-1β: SMD = −5.31, 95% CI (−6.09, −4.52), *p* < 0.00001].

## Discussion

AKI is a complex clinical syndrome with many possible causes and clinical symptoms. AKI is associated with high mortality and has an independent effect on the risk of death. Blood purification technology continues to improve, but the mortality of AKI patients has not decreased significantly, and a considerable number of surviving patients have progressed to end-stage renal disease, which requires long-term haemodialysis treatment, bringing heavy economic burden to society (Vijayan et al. 2021). The key to the treatment of AKI is to protect the renal function, which is likely to recover in a short period of time after active and effective treatment. Seising the window period of treatment is very important for the prognosis of patients with AKI. The mechanisms involved in the pathogenesis of AKI are complex, oxidative stress and inflammatory response play important roles in the progression of AKI. At present, there are few drugs to improve the renal function of patients with AKI, and the commonly used drugs in clinical practice are some Chinese patent drugs. Research of natural drugs and their single components for AKI shows a hot trend (Boozari and Hosseinzadeh 2017). In recent years, with the revelation of the antioxidation and anti-aging mechanism of RSV, the research on RSV has become more and more popular in kidney disease, especially in AKI (Galiniak et al. 2019). Many research funds and basic experiments have been invested in the research of RSV, which therapeutic effects cover many diseases and medical disciplines. Some experimenters have made benign results (Gu et al. 2021), but some researchers are not so lucky, because the research results on RSV are uneven, especially since there is not a unified public opinion on its therapeutic dose and maintenance time. The antioxidant mechanism of RSV has been confirmed in a large number of pieces of literature, but there is little relevant evidence about its relationship with the anti-inflammatory effect. The present systematic review and meta-analysis mainly intended to evaluate the anti-inflammatory properties of RSV when used in the treatment of AKI, RSV has a more beneficial effect on SA-AKI animal models than the others. The results showed that RSV was significantly associated with lower levels of TNF-α, IL-6, and IL-1β. Current evidence supports the anti-inflammatory properties of RSV.

Scr and BUN are the most important markers that reflect renal function status. Our meta-analysis suggested that RSV treatment was significantly associated with lower levels of Scr and BUN. Dose-response effects and time-response effects play an important role in clinical medication. However, no animal studies reported the dose-response effects and time-response effects of RSV when used in the treatment of AKI. In the meta-analysis reported here, with regard to Scr and BUN, the greatest effects were recorded in low-dose (<20 mg/kg/day) groups rather than in high-dose (>20 mg/kg/day) groups. Thus, it is necessary to notice that the dosage of RSV should be increased slowly starting from a low dosage in clinical use. Nevertheless, whether an excessive dose of RSV will suppress its therapeutic effects in the treatment of AKI should be further investigated. For time-response effects, subgroup analysis indicated that intervention duration of RSV can influence the treatment effect, and more beneficial effects were observed when studies had a drug administration time of <2 weeks. Therefore, it is difficult to determine the appropriate intervention duration based on current evidence. In multi-arm clinical trials, multiple levels of dosage or intervention duration are required to evaluate the dose-response effects or time-response effects and determine the optimal dosage or intervention duration.

Pro-inflammatory cytokines including TNF-α, IL-1β, and IL-6 are critical mediators in the development and progression of AKI. Our study suggested that RSV was significantly associated with a lower level of TNF-α, IL-1β, and IL-6. Based on the above results, there is no consensus about the anti-inflammatory property of RSV in the treatment of AKI. Therefore, the anti-inflammatory effects of RSV still need to be confirmed in more studies.

Several limitations should be considered in this systematic review and meta-analysis. First, a number of studies did not report baseline characteristics between experimental groups and control groups. Second, Egger’s test and asymmetry of funnel plots showed that publication bias existed, which could exaggerate the therapeutic effects of RSV. Therefore, the positive findings on RSV should be interpreted with caution. Third, some studies did not report a randomisation process.

## Conclusions

Our meta-analysis suggested that RSV treatment was significantly associated with lower levels of Scr and BUN. This result indicated that RSV has a beneficial effect on AKI animal models. The present systematic review and meta-analysis mainly intended to evaluate the anti-inflammatory properties of RSV when used in the treatment of AKI, RSV has a more beneficial effect on SA-AKI animal models than the others. The results showed that RSV was significantly associated with a lower level of TNF-α, IL-6, and IL-1β. Nevertheless, whether an excessive dose of RSV will suppress its therapeutic effects in the treatment of AKI should be further investigated.

## Author contributions

SC and ST designed the study. SC searched databases. SY collected the data. XF assessed the quality of the studies. SC and ST performed all analyses. SC wrote the manuscript. All authors contributed to this systematic review and meta-analysis.

## Data Availability

The original contributions presented in the study are included in the article, further inquiries can be directed to the corresponding author.
